# Development and application of an electronic synoptic report for reporting and management of low-dose computed tomography lung cancer screening examination

**DOI:** 10.1186/s12880-022-00837-y

**Published:** 2022-06-11

**Authors:** Alain Tremblay, Nicole Ezer, Paul Burrowes, John Henry MacGregor, Andrew Lee, Gavin A. Armstrong, Raoul Pereira, Michael Bristow, Jana L. Taylor, Paul MacEachern, Niloofar Taghizadeh, Rommy Koetzler, Eric Bedard

**Affiliations:** 1 Department of Medicine, Cumming School of Medicine, University of Calgary, 3330 Hospital Drive NW, Calgary, AB T2N 4N1 Canada; 2 Department of Medicine, McGill University Health Centre, McGill University, 1001 Decarie Blvd, Montreal, QC H4A 3J1 Canada; 3 Department of Diagnostic Imaging, Foothills Medical Center, Alberta Health Services, 1403 29 St NW, Calgary, AB T2N 2T9 Canada; 4 Department of Radiology and Diagnostic Imaging, University of Alberta, 2A2.41, 8440 112 St NW, Edmonton, AB T6G 2B7 Canada; 5 Department of Diagnostic Radiology, McGill University Health Centre, 1001 Decarie Blvd, Montreal, QC H4A 3J1 Canada; 6 Department of Surgery, Faculty of Medicine and Dentistry, Walter C. MacKenzie Health Sciences Centre, University of Alberta, Edmonton, 2J2.00T6G 2R7 Canada

**Keywords:** Radiology report, Lung cancer screening, CT scan, Synoptic report

## Abstract

**Background:**

Interpretation of Low Dose CT scans and protocol driven management of findings is a key aspect of lung cancer screening program performance. Reliable and reproducible methods are needed to communicate radiologists’ interpretation to the screening program or clinicians driving management decision.

**Methods:**

We performed an audit of a subset of dictated reports from the PANCAN study to assess for omissions. We developed an electronic synoptic reporting tool for radiologists embedded in a clinical documentation system software. The tool was then used for reporting as part of the Alberta Lung Cancer Screening Study and McGill University Health Centre Pilot Lung Cancer Screening Program.

**Results:**

Fifty reports were audited for completeness. At least one omission was noted in 30 (70%) of reports, with a major omission (missing lobe, size, type of nodule in report or actionable incidental finding in recommendation section of report) in 24 (48%). Details of the reporting template and functionality such as automated nodule cancer risk assessment, Lung-RADS category assignment, auto-generated narrative type report as well as personalize participant results letter is provided. A description of the system’s performance in its application in 2815 CT reports is then summarized.

**Conclusions:**

We found that narrative type radiologist reports for lung cancer screening CT examinations frequently lacked specific discrete data elements required for management. We demonstrate the successful implementation of a radiology synoptic reporting system for use in lung cancer screening, and the use of this information to drive program management and communications.

## Background

Lung cancer screening with low-dose chest computed tomography (LDCT) has now been demonstrated to reduce all cause as well as lung-cancer specific mortality in several clinical trials and is at various stages of implementation internationally [1–4]. Interpretation of LDCTs and protocol driven management of findings is a key aspect of program performance with impact on false positive rates, unnecessary invasive testing and overdiagnosis [5, 6]. A reliable and reproducible method to communicate the radiologist’s interpretation of the LDCT exam to the screening program or clinicians driving management decision would benefit such programs.

Radiology reports are typically of a narrative format dictated and then typed by transcriptionists or voice recognition software. Such narrative reports have been found to vary significantly between individuals and may lack completeness of specific details required for case and program management [7, 8]. Synoptic reporting of surgical, pathology and radiology reports have been shown to be more consistent and complete than narrative reports [7, 9, 10]. In addition, synoptic reporting contributes to creating structured databases, which facilitate data sharing and analysis for quality assessment, and future research [11]. Integration of radiology reporting with other protocol-based management and communication components of screening could also improve program efficiency. Electronic synoptic reporting of LDCT exams should be distinguished from classification and management systems such as Lung-RADS [12]. While such systems can be useful in reporting and standardizing recommendations, they are not in themselves synoptic reports and can still be used as part of a traditional didactic reports. Reporting templates have previously been proposed for LDCT reporting [13, 14]. Such structured reporting templates represent a form of synoptic reporting but lack many of the advantages of electronic software-based systems. This manuscript describes the development and application of such a system.

## Methods

### LDCT report audit

Fifty sequential baseline LDCT narrative type reports from the PANCAN lung cancer screening study [15]. Calgary site which enrolled in 2009 and 2010, starting at the 120^th^ participant, were reviewed by one of the non-radiologist investigators (AT). The reports were assessed for any major omission [defined as a reported nodule with missing lobe, size or type (solid, semi-solid, ground glass or peri-fissural) or missing inclusion of an actionable incidental finding in the impression section of the report] or minor omission (defined as a nodule missing a 2nd dimension measurement if > 3 mm, recording of scan slice number, nodule edge characteristic, presence or absence of emphysema and any comment on incidental findings or lack thereof). Image review was not performed, and as such the accuracy of information was not assessed, only its completeness.

### Development

A radiology synoptic template was developed by the investigators of the Alberta Lung Cancer Screening Study (ALCSS), which included respirologists, radiologists, thoracic surgeons, public health, and primary care specialists, all with prior experience with cancer and/or lung cancer screening. Softworks Group Inc (Edmonton, Alberta, Canada) was selected as a software platform vendor given their expertise and experience in synoptic reporting system and prior work within the Alberta Health Services health care environment. The web-based Synoptec™ clinical documentation system software was used as the host system. A preliminary reporting template was developed as a REDCap [16] instrument hosted by the University of Calgary Clinical Research Unit, with migration of data into the Synoptec system once it was ready to be activated. Ongoing changes to the reporting template were made as the program evolved and informal feedback received from the users.

LDCT scans performed as part of the ALCSS comprised most scans reported into the system, with additional cases from participants in the PANCAN [17] and Screening of Alberta Asbestos Exposed Workers for Lung Cancer and Mesothelioma studies. The studies were approved by the Health Research Ethics Board of Alberta (HREBA.CC-16-0496, HREBA.CC-16-0554 and HREBA.CC-14-0026) and all methods carried out in accordance with relevant guidelines. Written informed consent was obtained from all subjects participating in the above studies. The radiology reporting template is also linked to basic individual participant information entered in the Synoptec™ system including age, gender, smoking status, PLCOm2012 risk calculation, family history of lung cancer so that a nodule risk calculation (NRC) [18] can also be performed in real time as part of the report. Screening eligibility determination, scheduling of CT examination, queries for upcoming examination scheduling, missing results, or overdue examinations as well as data analytics for main program performance metrics can also be performed with the Synoptec™ platform.

A modified version of the template was developed for the McGill University Health Centre Pilot Lung Cancer Screening Program (REB MP-37-2019-5038). Key changes beyond formatting headers and text for the change in institutions were the use of Lung-RADS (v1.1) [12] rather than NRC as a driver of interpretation and recommendations. In addition, participant letters were generated in the French language as well as English so that the appropriate version could be selected when sending to participants.

### Statistical consideration

The results were summarized and reported using SPSS (IBM Corp. Released 2017. IBM SPSS Statistics for Windows, Version 25.0. Armonk, NY: IBM Corp). Descriptive statistics were applied with categorical variables presented as number and percent, whereas continuous ones were presented as mean and standard deviation.

## Results

### LDCT audit

Fifty didactic PANCAN study baseline LDCT reports initially interpreted by 4 different chest radiology trained sub-specialists were reviewed. At least one omission was noted in 30 (70%) of reports, with a major omission in 24 (48%). The most common major omission was missing nodule type (26/50, 53%), with one report missing any nodule measurement. The most common minor omission was lack of description of nodule edge characteristics or spiculation (25/50, 50%), missing comment on emphysema (11/50, 22%) and missing 2nd dimension size for a lesion (3/50, 6%).

### Reporting template

The electronic reporting template developed is divided in 4 main categories each on a separate page view: 1. Scan details and nodule information; 2. Incidental findings; 3. Auto-generated didactic type report; 4. Auto-generated participant results letter. A short video overview of the system is available.

**Fig. 1:**
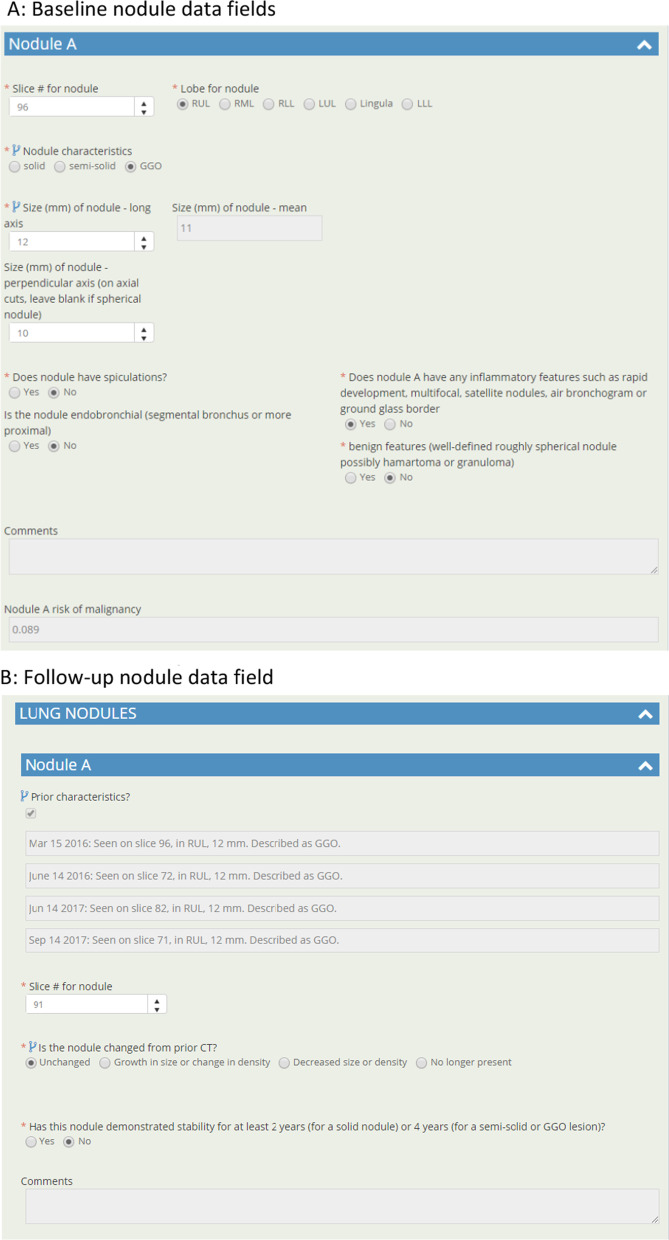
**A** Baseline nodule data fields. **B** Follow-up nodule data fields

**Fig. 2:**
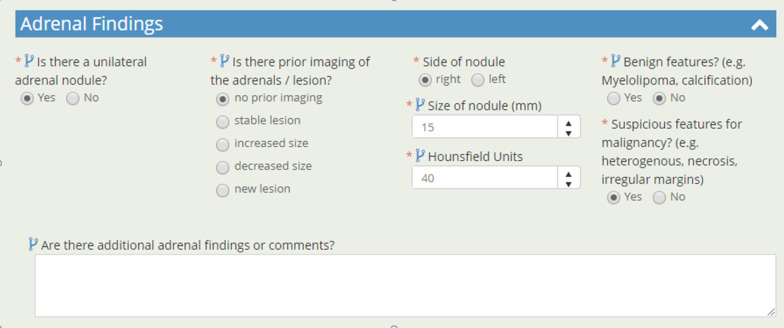
Adrenal nodule incidental finding data fields

**Fig. 3:**
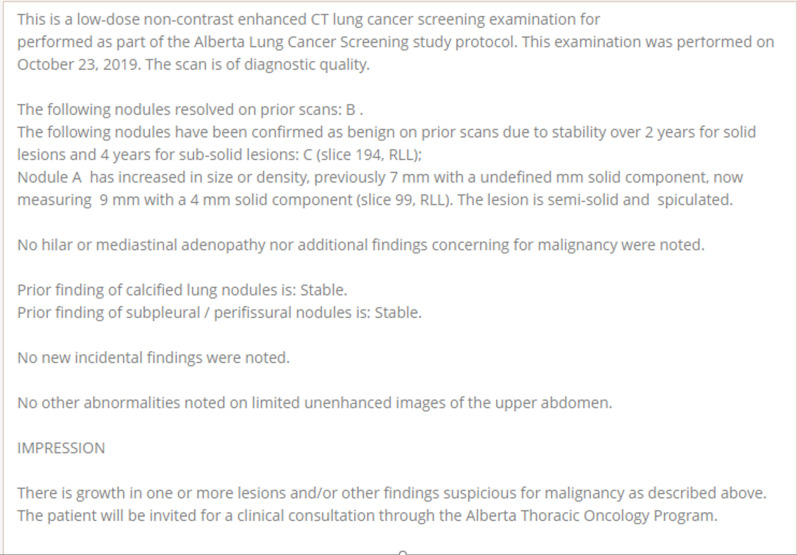
Auto-generated narrative report

**Fig. 4:**
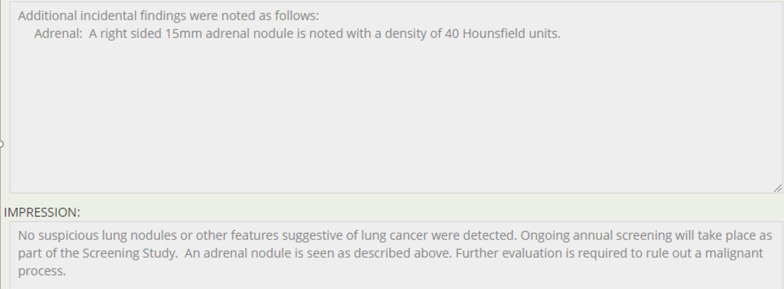
Auto-generated report with an actionable incidental finding

**Fig. 5:**
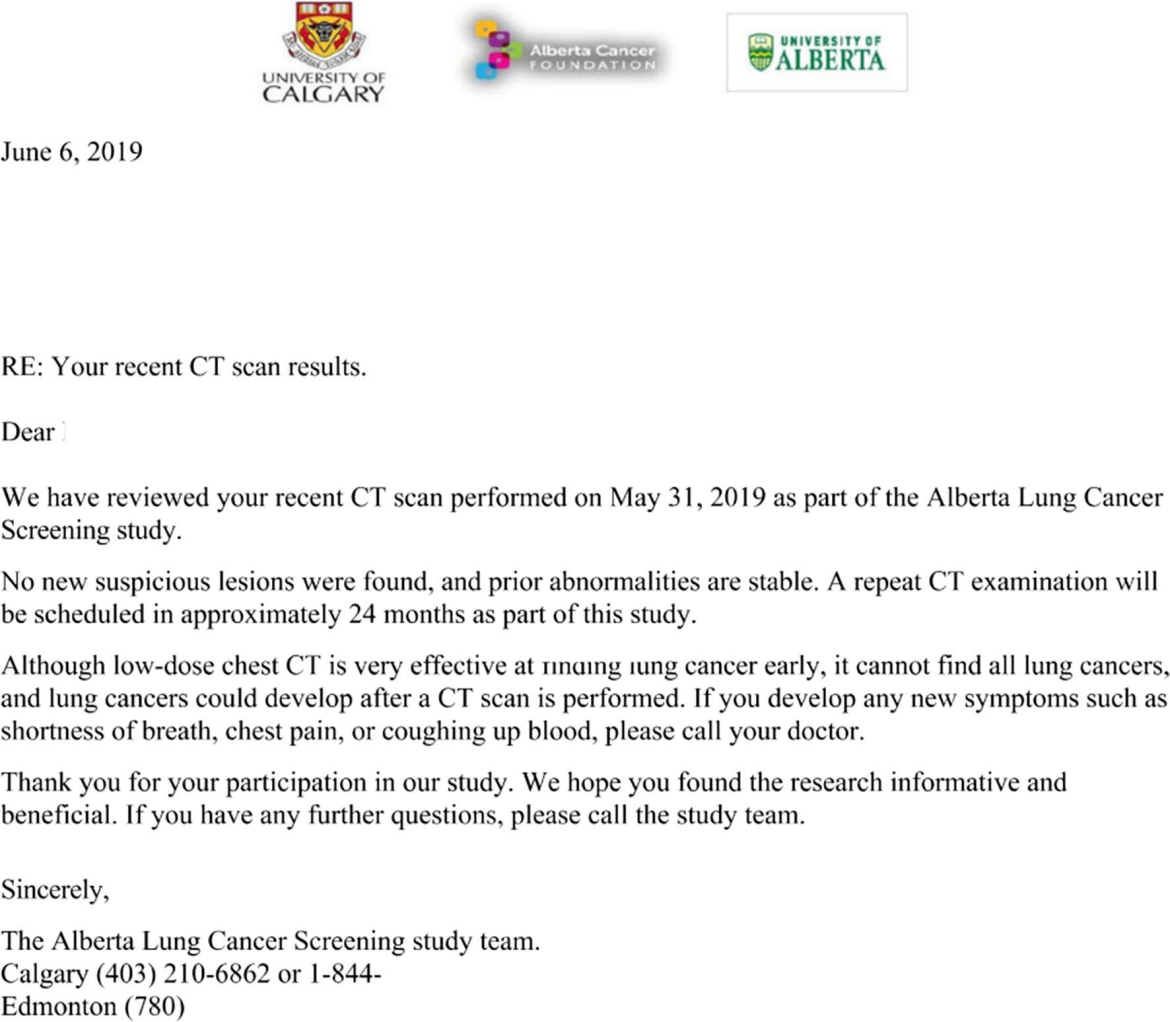
Auto-generated participant letter for a follow-up LDCT scan

1. *Nodule details* Radiologists can comment on any quality issues with the examination, and if the examination should be repeated/deemed incomplete. Presence of radiologic emphysema is recorded. On baseline scans, up to 5 nodules can be reported, and other new nodules added beyond 5 on subsequent scans, up to a maximum of 26. Required lesion information includes location (slice #, lobe and if proximal endobronchial), size (2 dimension), and presence of spiculations. Nodule malignancy risk calculation is presented in real time (Fig. 1A). On follow-up examination, prior nodule characteristics are brought forward into the report to aid with interpretation, and if unchanged only CT slice number needs to be entered (Fig. 1B). Nodules are confirmed as benign once stable for 2 (if solid) or 4 (if sub-solid) years. The highest risk nodule is highlighted by the system and any additional findings concerning for lung malignancy entered by the radiologist. This generates a clinical decision plan based on program parameters (this was based on the NRC in the ALCSS [6, 19]), which the radiologist can then approve the plan or offer an alternative recommendation to, or request a review by the program. If a lesion is larger than 8 mm, the radiologist can recommend a PET/CT as next evaluation step. Adaptive logic rules are in place so that only relevant information is requested of the radiologist and non-relevant data fields hidden from view.
2. *Incidental findings* Radiologist can enter any incidental findings but are asked to determine if a clinical assessment is required for each entry. Entries are organ-based, with common findings having checkbox entry options, with text box options for others. Specific synoptic datapoints are requested for certain common incidental findings (e.g. thyroid, adrenal nodules) so that automated recommendations can be generated based on the American College of Radiology White Papers on Incidental Findings [20, 21] (Fig. 2).
3. *Auto-generated didactic type report* The system uses the synoptic data to generate a narrative type of report more familiar to the clinicians receiving the report. This component is read-only but can be modified by the radiologist by changing entries in Sects. 1 or 2 above. The summary section includes actionable items and recommendations for the screening or clinical team receiving the report, including next screening scan interval, need for referral for clinical assessment and highlighting any incidental findings which may need further assessment (Figs. 3 and 4).
4. *Auto-generated participant results letter* The system uses the synoptic data to generate a lay-person narrative letter describing the results of the examination, including nodule risk range and any organ system with an incidental finding which warrants follow-up with their primary care provider. If emphysema is noted on the baseline scan, a note of this is also made. A reminder is included to remind participant to seek care for any new symptoms regardless of scan results, and to consider smoking cessation if identified as a current tobacco user at program entry. The letter is read-only for the radiologist but can be edited by the screening coordinator if needed before being sent to the individual (Fig. 5).

Once the report is signed, the report can be distributed though linkage of the Synoptec™ software with electronic medical records or PACS systems. Due to budget and time constraints, this integration was not implemented for our study, and a cut & paste of the report performed by the radiologists into their usual voice dictation window was then submitted as per the standard diagnostic imaging reporting process.

### Reports

In Alberta, 224 baseline CT scans were first reported in the REDCap reporting tool and subsequently imported into the Synoptec system. An additional 2,657 LDCT scans were reported directly into the Synoptec system for a total of 2881 reports. A total of 9 radiologists interpreted the examinations which were performed in 3 Alberta Hospitals and 1 outpatient private radiology clinic.

Of 836 baseline scans, 324 (38.7%) had reported evidence of emphysema which generated a comment in the participant results letter. In 200/302 (66.2%) cases where the information was available, the participant did not report a prior diagnosis of COPD or emphysema. At least one nodule was reported in 355/836 (42.4%) scans, with a mean of 2.8 nodules in those individuals and with 47 (5.6%) reporting the maximum of 5 nodules. Mean highest nodule NRC was 5.7% (SD ± 13.4%, range 0–82%). Other findings concerning for malignancy were noted in 7 (0.8%), in all cases consisting of mediastinal adenopathy. Almost all baseline scans had at least 1 incidental finding reported (815/836, 97.5%), on average 2 per examination, but only 187 (22.4%) were suggested to require a clinical evaluation.

Lung-RADS classification was generated for 2349 LDCT examinations (this feature was not developed for the initial phase of the project), with the distribution outlined in Table 1. The radiologists concurred with the program-based auto-generated management plan in 2633/2762 (95.3%) of examinations, and PET/CT was suggested in 36 cases. In 35 cases (1.3%) a more aggressive plan was suggested such as earlier CT, PET/CT or clinical assessment. In 27 cases (1%) a less aggressive plan was suggested such as no further follow-up required, or no need for clinical assessment or early recall prior to next 12-months screen. In other cases, comments related to non-nodule incidental findings or other comments which did not relate directly to the management plan. A total of 3619 nodule entries were performed, but in 3192 (88.2%) of assessments, the nodule was stable so that minimal data entry was required in such lesions. Lesions were smaller in 228 entries (6.3%) and increased in size or density in 126 (3.5%). In 569 instances, a prior nodule was no longer seen on review of a current LDCT.

**Table 1:** Lung-RADS classification for LDCT scans in Alberta and McGill screening projects

	Alberta		McGill	
	Frequency	Percent (%)	Frequency	Percent (%)
Lung-RADS category 0–Incomplete	15	0.6	0	0.0
Lung-RADS category 1	1129	48.1	20	12.7
Lung-RADS category 2	1007	42.9	118	74.7
Lung-RADS category 3	60	2.6	12	7.6
Lung-RADS category 4A	62	2.6	4	2.5
Lung-RADS category 4B	64	2.7	4	2.5
Lung-RADS category 4X	12	0.5	0	0.0
Modifier S	374	15.9	15	9.5
Total scans	2349		158	

For the McGill component of the project, 158 additional LDCTs were reported into the system with a total of 4 radiologists in 2 hospitals. Lung-RADS scoring for these scans is shown in Table 1.

Among the baseline scans 83/97 (85.6%) had reported evidence of emphysema. There was at least one nodule was reported in 78/97(80.4%) scans. Lung-RADS classification was generated for the 158 LDCT examinations, with the distribution outlined in Table 1. A higher percentage of Lung-RADS 3 lesions are noted in this cohort, likely due to the larger proportion of baseline vs. follow-up examinations. The radiologists concurred with the program-based auto-generated management plan based on the Lung-RADS classification in all examinations, and PET/CT was suggested in 1 patient.

## Discussion

We found that narrative type radiologist reports for LDCT lung cancer screening examinations frequently omitted specific discrete data elements that may be required for management decisions as part of a screening program. We developed an electronic synoptic reporting system for such exams supporting more complete reporting of findings and allowing real-time nodule risk malignancy prediction, exam classification according to this risk or by the Lung-RADS categories. While generating discrete and analyzable data points, the system generates a report as well as participant results letter in a readable format familiar to the target user. We then applied the system successfully in the interpretation of a large number of LDCT examinations by multiple radiologists in several hospitals as part of 2 separate lung cancer screening projects.

The audit of narrative reports highlighted that this approach frequently leaves out discrete information items. This is not meant as a criticism of the work performed by the radiologists. In fact, just as in regular conversation and reading, the radiologist and by extension an expert clinician reading the report will often infer information that may be explicitly lacking. For example, a nodule will likely be assumed to be solid unless noted to be otherwise, and round if only one dimension is stated. But while this approach may be practical for individual patient management, protocol driven large volume screening programs will likely function best with explicit documentation of all relevant characteristics. Details for smaller nodules may not be seen as relevant by the radiologist or even possible to determine and may not represent a true deficiency as far as clinical decision making is concerned. Program expectations in the PANCAN study may also have paradoxically reduced data reported in the clinical reports as the radiologists were also asked to complete a paper-based study report form with all of the relevant metrics included (e.g. reporting emphysema). This may have reduced radiologists’ effort to include as much information in the dictated report, although they were aware that this report and not the study forms would stand as the report of record in the patient medical file.

Our finding of missing data from narrative reports is consistent with published evidence. For example, one study found that less than 50% of key staging elements were included in cancer staging radiology reports, whilst this increased to 87.3% with a standard reporting template [9]. Other groups have described the development of a synoptic template specifically for lung cancer screening, but not its implementation [13, 14]. Nodule reporting tools have also been described which can improve completeness of follow-up recommendations for incidentally found lesions [22]. Our finding that the radiologist agreed with the automatically generated management plan in > 95% of cases suggests that protocols based on the synoptic data can be an efficient and reliable method to drive management of screened individuals in such a program.

We did not capture data on any efficiency metrics for the reporting radiologists, although other authors have documented decreased completion time for synoptic type reporting [23]. Anecdotally, the change in practice at the onset created inefficiencies. As experience with the system improved, and built-in assistance in reporting follow-up LDCT scans by automatic transfer of prior nodule information into the report became more relevant, our radiology group embraced the system. For scans with significant unexpected findings, more flexibility in modifying report recommendations and direct integration of voice dictation functionality could have been of benefit while such modifications were possible in the reporting system, this required keyboard typing and it was not always intuitive if and where an added text entry would appear in the auto-generated report. We do recommend that synoptic system retain some degree of flexibility allowing radiologists to add clarifications, justifications and recommendations beyond what is included in the standardized reports.

Value added aspects to the system included automatically generated letters for communication of results to participants, including information regarding lung nodules, actionable incidental findings, emphysema, smoking cessation and next recommended LDCT examination. As well, program volumes and quality metrics such as early recall rates can be tracked as needed for program monitoring and reporting, and raw data exported as needed for more detailed statistical analyses.

Our report is limited in that we did not simultaneously compare the synoptic reporting process to the standard narrative reports. To do so in a single center is difficult, as the reporting radiologists would likely “learn” the data points of interest through synoptic reporting, likely impacting how narrative reports are generated. The system described is not a computer aided detection (CAD) tool in that no image analysis is made by the software. Future integration of CAD systems with reporting and screening management could further improve the screening process. In addition, there is a need for conducting semi-structured qualitative interviews to assess the prospects of radiologists and data abstractors to determine the potential barriers to implementing of the proposed strategy. Formal assessment of user satisfaction could also be considered. Cost of development and implementation of such systems as well as integration with current electronic medical records may represent additional barriers.

## Conclusions

In conclusion, we demonstrate the successful implementation of a radiology synoptic reporting system for use in lung cancer screening, and the use of this information to drive program management and communications.

## Data Availability

The datasets generated and/or analysed during the current study are not publicly available due them being drawn from other primary studies, but these may be available from the individual study primary investigators on reasonable request by contacting the corresponding author.

## Abbreviations

ALCSS: Alberta lung cancer screening study
CAD: Computer aided detection
LDCT: Low-dose computed tomography
NRC: Nodule risk calculation
